# Sublimate-Paper as a Surgical Dressing

**Published:** 1888-01

**Authors:** 


### *SUBLIMATE-PAPER AS A SURGICAL DRESSING.*

Filtering-paper, which has been soaked in a solution of corrosive sublimate, 1 to 500, with the addition of glycerine, five per cent., and then dried, is used with excellent results as a dressing. It has been proven valuable, not only in small, superficial wounds, but also in some important operations, such as extirpation of cervical glands, amputation of fingers, and even in a case of amputation at the upper part of the thigh, in which the first dressing, after careful approximation of the edges of the wound, remained unchanged for ten days. Gedeke uses the sublimate-paper folded in from two to eight thicknesses, according to the necessities of the wound, and bandages the dressing to hold it in position.

As a result of his observations, he comes to the following conclusions:

1. That filtering-paper, thoroughly soaked in a solution of corrosive sublimate of the strength of 1 to 500, makes a good material for dressings.

2. For a dressing, it is necessary, according to the extent of the wound, to use the paper in two to eight layers, and to complete the dressing by a dry bandage exterior.

3. This method is pre-eminently suitable for fresh wounds.

4. In complicated injury of the fingers, the dressing has the advantage of rendering the fingers immobile.

5. It should not be left on longer than two or three days.

6. In case no antiseptic dressing is ready, the sublimate-paper can be used for a short time to make a suppurating wound aseptic.—*Centralblatt für Chirurgie.*

